# Ecological distribution conflicts as forces for sustainability: an overview and conceptual framework

**DOI:** 10.1007/s11625-017-0519-0

**Published:** 2017-12-13

**Authors:** Arnim Scheidel, Leah Temper, Federico Demaria, Joan Martínez-Alier

**Affiliations:** 1 International Institute of Social Studies (ISS), Erasmus University Rotterdam (EUR), The Hague, The Netherlands; 2 Institute of Environmental Science and Technology (ICTA), Universitat Autónoma de Barcelona (UAB), Barcelona, Spain

**Keywords:** Environmental justice, Social movements, Social metabolism, Sustainability transitions, Grassroots politics, Environmental Justice Atlas

## Abstract

Can ecological distribution conflicts turn into forces for sustainability? This overview paper addresses in a systematic conceptual manner the question of why, through whom, how, and when conflicts over the use of the environment may take an active role in shaping transitions toward sustainability. It presents a conceptual framework that schematically maps out the linkages between (a) patterns of (unsustainable) social metabolism, (b) the emergence of ecological distribution conflicts, (c) the rise of environmental justice movements, and (d) their potential contributions for sustainability transitions. The ways how these four processes can influence each other are multi-faceted and often not a foretold story. Yet, ecological distribution conflicts can have an important role for sustainability, because they relentlessly bring to light conflicting values over the environment as well as unsustainable resource uses affecting people and the planet. Environmental justice movements, born out of such conflicts, become key actors in politicizing such unsustainable resource uses, but moreover, they take sometimes also radical actions to stop them. By drawing on creative forms of mobilizations and diverse repertoires of action to effectively reduce unsustainabilities, they can turn from ‘victims’ of environmental injustices into ‘warriors’ for sustainability. But when will improvements in sustainability be lasting? By looking at the overall dynamics between the four processes, we aim to foster a more systematic understanding of the dynamics and roles of ecological distribution conflicts within sustainability processes.

## Introduction

Transitions towards more sustainable futures could benefit from supporting those civil society actors that relentlessly oppose and transform local unsustainabilities across the globe. Instead, persecution, criminalization and violence against such grassroots activists, including their brutal assassination are increasing (Del Bene et al. this feature; Navas et al. this feature). Global Witness (2017), for instance, recently reported that 200 environmental defenders have been killed in 2016. Many of these civil society actors turned into environmental activists to contest cases of unsustainable extraction, trade and consumption of resources, because these activities threatened their own livelihoods. Gadgil and Guha (1995) have called those who resist environmental devastation to defend their own livelihoods ‘ecosystem people’ and Martinez-Alier (2002) has referred to them as ‘environmentalists of the poor’. In their acts of resistance, they contribute to a larger social purpose—by not only opposing and sometimes transforming unsustainable resource uses, but also by creating needed political debates on the use of the environment, and by constantly renegotiating public values of what is considered ‘sustainable’. Often criminalized by governments and companies for their actions, we argue that such activism is among the most promising social forces to promote not only social justice but also environmental sustainability. They might be seen as an example of Polanyi (1944)’s double movement, meaning a self-protection of society against the commodification of life and nature.

Addressing issues of justice is a fundamental component of sustainability science (Jerneck et al. 2011; Golub et al. 2013). Understanding the ways how ecological distribution conflicts and environmental justice movements can contribute to both social justice and environmental sustainability is, however, not straightforward. It requires asking *why, through whom, how and when* do conflicts over the use of the environment take an active role in shaping transitions toward sustainability. Answers to these questions can be found in studying the processes through which unsustainable resource uses have given rise to ecological distribution conflicts and environmental justice movements, as well as the pathways that such movements have taken to transform them (Temper et al. 2018). Empirical research linking changes in resource uses and social metabolism, society’s processes of extraction, trade and disposals of material and energy, to the rise of ecological distribution conflicts, have grown over the past two decades (Martinez-Alier 2002; Martinez-Alier et al. 2010; Muradian et al. 2012). As has the body of empirical studies on environmental justice movements that have emerged out of such conflicts, fighting to protect not only their livelihoods but also the environment surrounding them (e.g. Pellow et al. 2002; Temper et al. 2015; Martinez-Alier et al. 2016). The way these processes of social metabolism, ecological distribution conflicts, environmental justice movements and transitions towards sustainability interact with each other can be multi-faceted, requiring nuanced research on each of these interactions. Yet to better understand the broader dynamics at play calls for a conceptualization of the interactions of all these processes as a whole, in a systematic way. So far this has not been done.

This overview paper, therefore, presents a conceptual framework that schematically maps out and describes the dynamics of interaction between social metabolism, ecological distribution conflicts, environmental justice movements, and sustainability transitions. For scholars new to the field, we aim to review and summarize some of their key linkages. For the advanced study of the role of environmental justice movements within sustainability processes, we aim to push further an understanding of the overall dynamics at play between social metabolism, environmental conflicts, grassroots politics and sustainability transitions. By drawing these research fields together, we revisit and identify key arguments regarding *why, through whom, how and when* ecological distribution conflicts can play a role for sustainability and illustrate them with empirical examples and insights from the Atlas of Environmental Justice (EJAtlas, http://www.EJAtlas.org). While we acknowledge that pathways of conflict and resistance are ‘anything but a foretold story’ (Alonso-Fradejas 2015), we are particularly interested in scoping and reviewing those processes that can contribute to sustainability transitions.

As we argue, unsustainable resource uses create not only environmental destruction, but also conflicts and social forces that contest them, as seen in the 2200 cases registered in the EJAtlas by August 2017. Ecological distribution conflicts relentlessly bring to light unsustainable resource uses affecting people and the planet as well as conflicting values over the environment. Environmental justice movements, born out of such conflicts, can become, therefore, key actors in politicizing and confronting such unsustainable resource uses, by pushing public debates on the use of the environment, and also through formal means of contestation, and through direct and disruptive actions to stop unsustainabilities. We argue that such politically contentious actions can be very effective in enhancing ecological sustainability and social justice. By examining the overall dynamics between resource use patterns, conflicts, and mobilizations, we present some reflections on the conditions required for lasting sustainability transitions and the role environmental justice movements may play in these.

The next section introduces the conceptual backgrounds upon which we build our analysis: social metabolism, ecological distribution conflicts, environmental justice movements and sustainability transitions (“Concepts: social metabolism, ecological distribution conflicts, environmental justice movements and sustainability transitions”). We then move on to address some of the key relations between them, particularly in relation to sustainability issues (“From ecological distribution conflicts to sustainability transitions: understanding dynamic interactions”). Section “Breaking the vicious cycle of unsustainabilities and ecological distribution conflicts” focuses on their dynamics as a whole, and across different resource use regimes. Section “Conclusion” concludes on the role of environmental justice movements in shaping and repoliticizing sustainability processes.

## Concepts: social metabolism, ecological distribution conflicts, environmental justice movements, and sustainability transitions

### Social metabolism and socio-metabolic configurations

Sustainability depends largely upon the interactions and the material and energy exchange processes of socio-economic systems with the environment and its biogeochemical cycles. In this context, the concept of social metabolism has turned into a key approach to study such biophysical interaction processes. It originated from the idea that socio-economic systems—similar to biological organisms or ecosystems—require a continuous throughput of energy and materials to self-organize and to maintain and develop their internal functions and structures (Giampietro et al. 2014)^1^. Nowadays, social metabolism has become an interdisciplinary concept for which different applied methods have become available. They allow characterizing and quantifying the material and energy exchange processes for specific socio-economic processes as well as different types of societies (for an overview see Gerber and Scheidel 2018). Different societies have obviously distinctive metabolisms that sometimes co-exist and evolve over time. Compare, for instance, the material basis and forms of organization of hunter-gatherer, agrarian subsistence communities or industrial societies (Fischer-Kowalski and Haberl 2007). Their socio-metabolic characterizations allow not only understanding the very distinct sustainability problems faced by different societies in material terms (ibid), but also how resources are unequally allocated and consumed within and across them (Jerneck et al. 2011).

Beyond its biophysical dimension, society’s metabolism is also fundamentally characterized and shaped by *social, political and economic dimensions*, i.e. the political economy and the institutions of societies, which govern modes of appropriation, distribution and disposal of materials and energy. For instance, modern capitalism is among the main drivers of the current growth in social metabolism across the globe (Muradian et al. 2012) that furthermore defines substantially the social relations under which resources are extracted, used and disposed. In fact, capital accumulation takes place not only by expanded reproduction (i.e. the production and capitalization of new surplus value created by wage-labor) but also via extra-economic means, namely dispossession (i.e. the separation of the laborers from their means of production) (Harvey 2004), or contamination (i.e. the socialization of costs, or cost-shifting) (Demaria and D’Alisa 2013). Such processes further characterize the social metabolism. Following Demaria and Schindler (2016), we propose to use the term ‘socio-metabolic configurations’ to refer to both biophysical and social aspects of society’s metabolism. For instance, the metabolization of waste in Delhi, India has to do with the production, throughput and processing of waste (see EJAtlas 2014a). The materiality relates to the quantity, composition and calorific value of waste processes within the waste sector and its physical trajectory and transformation. The political economy has to do with how, where, and by whom it is managed, what is deemed to be waste, the forms of value attached, and the interests, laws and institutions that govern it. To understand how social metabolism relates to ecological distribution conflicts, one must not only look into the quantification and distribution of biophysical flows, but also upon the power relations that configure them. Finally, the co-evolution of its biophysical and social dimensions transforms and shapes resources uses. We refer to this as a political ecology of social metabolism.

### The study of ecological distribution conflicts

The term ‘ecological distribution conflicts’ emerged in the 1990s. It was coined by Martinez-Alier and O’Connor (1996) to describe social conflicts arising over the unequal distribution of environmental benefits, such as access to natural resources, fertile land, or ecosystem services, as well as over unequal and unsustainable allocations of environmental burdens, such as pollution or waste. By social conflict, we refer to a clash of interests, values and norms among individuals or groups that leads to antagonism and a struggle for power. From a Marxist perspective, such conflict constitutes the driving force of social life, with an emphasis on class struggle for ownership of the means of production. Further, we share the functionalist perspective of Simmel (1904) that considers how conflict can lead to the creation of new norms and institutional structures (see Temper et al., this feature, for further elaboration on conflict as transformative).

In contrast to ‘economic distribution conflicts’ over salaries, prices, profits or rents, ecological distribution conflicts cannot necessarily be resolved through economic measures, such as monetary compensation, or ‘correct price’ schemes, that would include internalization of social and environmental costs. These conflicts express themselves as struggles over valuation processes in terms of which are the values deemed relevant for decision making in particular projects, such as market and monetary values; livelihood values; indigenous territorial rights; or ecological values in their own units of account. For instance, can sacredness of a landscape imply a veto power over profit-oriented extractive industries (Temper and Martinez-Alier 2013)?

Research on ecological distribution conflicts has grown notably (Martinez-Alier 2002; Martinez-Alier et al. 2010; Muradian et al. 2012), whereas the term is often used interchangeably with similar notions of ecological, environmental, or socio-environmental conflicts (see Walter 2009). As the term suggests, the study of ‘ecological distribution conflicts’ puts particular emphasis on *distributional* aspects (who gets what environmental benefits and burdens) and related distributional justice claims. This does not mean that conflicts over procedural issues or recognition of different values and worldviews (Schlosberg 2004) are not considered. However, we consider that such conflicts are bivalent or trivalent in that they also often inevitably entail a distributional perspective, that is, the lack of participation and recognition contributes to unjust distributional outcomes. Environmental justice movements emerge out of ecological distribution conflicts, and claim just sustainabilities, simultaneously addressing environmental quality and human equality (Agyeman et al. 2003).

### The rise of environmental justice movements

In philosophy and ethics, ‘environmental justice’ pertains to the field of theories of justice that focuses on the natural environment. It includes debates on intergenerational equity and on the fair treatment of non-human species. In political ecology and environmental sociology, it focuses largely on the present generation, and the words ‘environmental justice’ apply to a social movement that has a precise date and place of birth: the United States in the early 1980s (Bullard 1990, 1994; Pellow et al. 2002). This movement, with roots in the Civil Rights movement, defended ‘people of color’ against environmental and health damage. The concept arose because minority communities were seen as being disproportionately subjected to higher levels of environmental burdens, which led to the emergence of a grassroots campaign against environmental racism and for environmental justice, spearheaded by activists including religious leaders.

Such concepts were later taken up by environmental sociologists and geographers. Parallel to the establishment of political ecology as an established academic field beginning in 1987 (Blaikie and Brookfield 1987) and its focus on the Global South (Peet and Watts 1996; Bryant and Bailey 1997), the US environmental justice movement from the outset was concerned with justice beyond the US. In 1990, it proclaimed the 17 Principles of Environmental Justice in a meeting in Washington DC, focusing on damage to minority groups in the US and making also a strong appeal for all peoples of color in the world to rise against ‘environmental racism’, and calling for respect for other species^2^. Nowadays a global movement for environmental justice is flourishing with greater strength than in the US, although often subject to strong repression. The movement has emerged out of worldwide struggles against open-pit mining, fossil fuel extraction, tree plantations, dams, nuclear energy, waste disposal, urban pollution and other issues, as the over 2200 cases gathered in the EJAtlas testify to^3^.

The actors of such movements are comprised not only of those directly affected by one project. They often involve affected communities elsewhere, or activists and organizations not directly affected but conscious about the caused environmental destruction, who empathize with affected groups and who aim to change the larger power structures leading to systematic unjust distribution of environmental benefits and burdens. Through such alliances, mobilizations against unsustainabilities can go beyond ‘Not In My Backyard’ (NIMBY) concerns limited to specific places. They build the basis for larger movements that question the broader structures causing environmental injustices. Their approach is often radical and broad-minded. For instance, they might reaffirm the rights of affected people, such as workers or indigenous, to safety and health, oppose capitalism and the destructive operations of multi-national corporations as a central cause of environmental injustices, and at the same time declare the sacredness of Mother Earth (Temper et al. 2015; Martinez-Alier et al. 2016).

### Visioning sustainability transitions

The aim to radically restructure current systems of production, consumption and exchange are also shared by the flourishing literature on ‘sustainability transitions’ (Grin et al. 2010; Brown et al. 2013). This term refers to a growing consensus that holds that the pervasive and wicked environmental challenges humanity faces differ in scope, scale and complexity from previous environmental challenges and call for responses that go beyond incremental changes or new technologies. Sustainability transitions are meant to be different from quick techno-managerial ‘sustainability fixes’. For example, closing one polluting factory is a one-time fix, whereas establishing and enforcing laws that prohibit polluting factories is a transition, reflected in actions that may augur a broader transformation in the regime of production.

One branch of scholarship on transitions, rooted in innovation and Science and Technology Studies, primarily aims to understand historical technological change and how the development of specific technologies and institutional frameworks lead to the reconfiguration of socio-technical relationships (Geels 2010). Stemming from this understanding of the factors which enable or constrain transitions, transition management is a policy-oriented application of transition theory that seeks to guide society towards more sustainable futures (Kemp et al. 2007). While transition theories are inherently normative, in that they call for radical systemic shifts in deeply held values and beliefs, patterns of social behavior (Westley et al. 2011); the field has come under critique for being depoliticized, managerial and limited in its analysis of the deeply political and contested nature of sustainability transitions (Shove and Walker 2007; Stirling 2015; Avelino et al. 2016).

There is space for further engagement between transitions studies and critical perspectives from political ecology, social movement theory, critical environmental justice studies (see for instance Geels 2006; Lawhon and Murphy 2012), as well as with voices both within and beyond the academy advocating for more radical transitions (Escobar 2015), sometimes referred to as ‘transformations’ (Temper et al., this feature). They include degrowth (see special feature in this journal, Asara et al. 2015); post-capitalism (Gibson-Graham 2006); radical ecological democracy (Kothari et al. 2015); or buen vivir (Gudynas 2011). These are often meant to be alternatives to (and not of) development, and intend to outline that there is politics beyond a unilinear future, unsustainable and unjust, consisting primarily of economic growth (Kothari et al. 2015). We suggest that a systematic view on the role of ecological distribution conflicts and environmental justice movements in sustainability transitions can provide meaningful inputs to understanding how such transitions happen. This is precisely what we address in the next section.

## From ecological distribution conflicts to sustainability transitions: understanding dynamic interactions

**Fig. 1:**
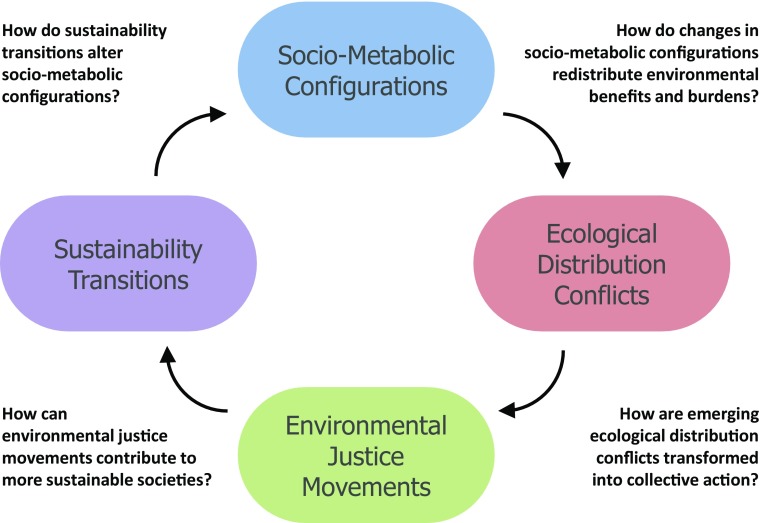
Schematic overview and key questions to understand interactions between socio-metabolic configurations, ecological distribution conflicts, environmental justice movements, and sustainability transitions. Source: the authors

How do the above introduced processes and patterns of socio-metabolic configurations, ecological distribution conflicts, environmental justice movements and sustainability transitions shape each other? While there are numerous interactions and outcomes between them, we particularly focus in this section on those relevant within sustainability processes. Figure 1 shows a schematic overview of their interactions and related key questions.

### Changes in socio-metabolic configurations redefine distribution of environmental benefits and burdens

Research on the links between social metabolism and ecological distribution conflicts has generally focused on how increases or changes in the former provoke conflicts by causing unjust and unsustainable allocations of environmental benefits and burdens. Burdens sometimes take the form of market ‘externalities’ (or else, cost-shifting), such as pollution from extractive industries (e.g., Teran, 2017). They often also include dispossession and displacement of people to make way for extractive industries (Martinez-Alier 2002; Martinez-Alier et al. 2010; Muradian et al. 2012). For instance, Martinez-Alier identified a “three-tier relation between the increasing social metabolism of human economies pushed by population and economic growth, the resulting ecological distribution conflicts among human groups, and the different languages of valuation deployed historically and currently by such groups when they reaffirm their rights to use the environmental services and products in dispute” (Martinez-Alier 2009).

The hypothesis of ‘more metabolism, more conflicts’, most fruitfully applied to national economies (e.g., Perez-Rincon et al., this feature), is a difficult one to test. While there are clear (read increasing) historical trends on material flows (Schaffartzik et al. 2014), this would need to be compared with historical and exhaustive inventories of ecological distribution conflicts. The EJAtlas represents such an inventory, with 2200 cases globally by August 2017, but still this remains an uneven sample of an unknown total across countries. Further, there are numerous other (local) factors influencing whether conflicts will emerge and the characteristics they may take. These are for instance the pace of environmental change at given scales: fast or slow, and the ability to establish a connection between socio-metabolic changes and environmental and health impacts and the capacity of actors to adapt to these changes in a way that they perceive as just. For example, climate impacts related to carbon emissions may still not be identified as such by many actors suffering from weather disruption. Also changes in the composition of material flows extracted from the environment, usually accompanied by changes in the actors extracting them, matter. For example, farming communities sustainably extracting biomass displaced by a mining project extracting minerals will protest because of a clash between two incompatible socio-metabolic configurations (Silva-Macher and Farrell 2014). Finally, also the direction and dynamics of change influence whether conflict emerges or not. For example, we may assume that increased extraction of a mining project will lead to conflicts with neighbors due to increased pollution. Yet in some situations also decreases in material extraction can cause conflicts. For instance, forest conservation policies that established the Sri Nakarin Dam National Park, Thailand, posed a ‘moratorium’ on forest biomass extraction, i.e. firewood or non-timber forest products (NTFPs), which strongly affected the livelihoods of forest-dependent communities (EJAtlas 2015a). To these examples of biophysical dynamics influencing conflict outcomes, we further need to add political, social and institutional aspects of metabolism affecting distributive aspects, i.e., how it is governed and shaped by power relations across its stages of extraction, distribution and disposal (Demaria and Schindler 2016).

To understand the full spectrum of how social metabolism relates to ecological distribution conflicts, the central question is *how changes in socio-metabolic configurations redefine the distribution of environmental benefits and burdens across different actors, therefore creating unjust distributional outcomes that give rise to distributional conflicts*. An overall increase in social metabolism (nationally or globally) may indeed alter all the above-mentioned factors, of which many address the local scale, thereby reconfiguring distributional outcomes. To this broad hypothesis of ‘more metabolism, more conflicts’ focusing on quantitative aspects (i.e. size of total material flows, number of conflicts across stages of production, transport and disposal), we also emphasize the role of qualitative material aspects: ‘the more ecologically harmful, the more socially conflictive’. To take the example of nuclear waste, only small amounts of such toxic materials will lead to conflicts over their allocation. However, since nuclear waste problems can also be seen in light of overall increases in societal energy demand, the first hypothesis still holds in this case.

Summing up, both large and ecologically harmful levels of social metabolism are generally characterized by intensive pollution/environmental destruction at the frontiers of extraction, processing and disposal. Changes in the social metabolism often imply new environmental burdens which are disproportionately allocated to some social groups, creating unjust distributional outcomes that may turn into visible conflicts. Returning to the question of ‘why’ ecological distribution conflicts play a role for sustainability, we argue that they fundamentally expose such unsustainable resource uses, by putting them into the spotlight. As discussed next, conflicts hold tremendous power for change by mobilizing social forces that can contest, politicize and transform such unsustainabilities.

### Ecological distribution conflicts mobilize environmental justice movements

Ecological distribution conflicts have given rise to many environmental justice movements around the globe. An illustrative example is the case from Kōchi, Japan, during the 1970s, where after decades of air and water pollution, citizen and fishermen groups initiated a movement to remove a paper pulp factory. When the company management refused to negotiate with the citizens group in May 1971, the group resorted to direct action by pouring cement into the mouth of the factory effluent outlet. Being under pressure, the administrative authorities were forced to ask the company to either move the factory elsewhere or to install proper pollution-control equipment. The company was unable to meet these demands and closed the polluting factory in May 1972 (see EJAtlas 2016a). Globally, around 17% of all environmental conflicts registered in the EJAtlas report environmental justices ‘successes’, such as stopping an unsustainable project^4^.

The answer to our initial question of ‘who’ are the actors through which ecological distribution conflicts most directly can shape sustainability processes is simple: it is through environmental justice movements, comprised of those most directly affected by such unsustainabilities and those allying with them. However, to explain when and how strong environmental justice movements emerge, we need to ask *why do some cases of unsustainable, unjust ecological distribution give rise to successful environmental justice movements, and why others not?* This question fundamentally aims to understand the conditions under which affected actors have been able to enact (successful) collective action against environmental injustices. It represents one of the core inquiries of (environmental) social movement studies (e.g. Keck and Sikkink 1998; McAdam et al. 2001; della Porta and Rucht 2002; Heijden and van Der 2006).

The concept of ‘political opportunity structures’, understood as the characteristics of a political system that facilitates or constrains collective action, has been key to understand strategies, successes, organization and mobilization levels of movements (Heijden and van Der 2006). Analyzing such political opportunity structures is important for understanding the venues chosen for successful lobbying and political actions. Movements further build up their ‘repertoires of contention’ in terms of protest forms and direct actions, which are often shaped by national and local contexts and histories (Tilly 2002). Timing and proactivity of collective action is also a key to achieving environmental justice. The EJAtlas demonstrates that the sooner mobilization occurs, the more likely success is. For instance, out of the almost 380 EJAtlas cases reported as an environmental justice success (such as ‘project stopped’), 57% of cases involved preventive mobilizations, whereas those with the mobilization beginning only in reaction to construction/operation represent 27%, and those where mobilizations arise in response to damages only 13%^5^.

In environmental justice struggles, the biophysical characteristics of the conflict can further shape the forms of mobilization and direct action. Resistance strategies can take advantage of ‘biophysical opportunity structures’, where they attempt to identify, change or disrupt the damaging ecological processes they are confronting towards their cause. Consider for instance pulling out of saplings to halt tree plantations, as has been the case in protests against eucalyptus plantations, in Tumkur, Karnataka, India (Gerber 2011; EJAtlas 2014b), uprooting of genetically modified crops, burning of wood logs to oppose illegal logging (EJAtlas 2015b), or countless cases of land occupation by the landless.

Finally, the ‘collective action frames’ (Tarrow 1992) of movements emerging in response to environmental conflicts becomes very powerful when they challenge current understandings of our relationships with the environment. These frames are often expressed through pithy protest slogans, that we refer to as the ‘vocabulary of environmental justice’ and which includes concepts and phrases such as ‘environmental racism’, ‘tree plantations are not forests’, ‘keep the oil in the soil’, ‘keep the coal in the hole’ and the like (Martinez-Alier et al. 2016). Such concepts and slogans draw on a collective identity of those negatively affected by ecological distribution conflicts. By offering a new vantage point, they aim to reframe and create new environmental narratives that resonate with the public and open the potential for broader alliances. They serve thus as mobilizing frames.

Pellow et al. (2002) emphasized the following key points to understand the emergence of environmental justice movements: (a) the importance of the history of environmental inequalities and the processes by which they unfold. This entails taking into account longstanding liabilities, as well as future concerns in environmental policy-making. (b) The role of social stratification by ethnicity, race, class (and caste), given the fact that the poor and people of color are generally the most vulnerable to environmental inequalities. These are not ‘minorities’—they are the majority of humankind, if not the ‘99%’. However, it must be kept in mind that communities and racial groups are frequently divided, as addressed in the next point. (c) The role of multiple stakeholders in these conflicts and their internal divisions. An analysis of the political dynamics within and between movements, based on understanding the different interests of classes, social identities and ideologies helps to understand current frictions as well as possible alliances to strengthen movements (see Edelman and Borras 2016). (d) The role of marginalized groups in reshaping environmental inequalities. For example, indigenous people and ethnically discriminated groups are involved in 44% of the EJAtlas cases. With their territories located at the frontiers of resource extraction, they often take a leading role in mobilizations, but also face disproportionately high rates of repression, including murder (see Del Bene et al. this feature; Global Witness 2017). Also the role of women leaders is noticeable in many environmental justice conflicts worldwide. It is often the marginalized segments of society who shape the contours of environmental justice struggles.

### Environmental justice movements can support sustainability transitions in various ways

The environmental justice perspective unmasks the questions of ‘who gets what environmental goods and bads, why, and in what amounts’, calling for grassroots movements to struggle for environmental health strategies to ensure the equal protection of all citizens, including indigenous peoples who often live at the extractive commodity frontiers. For instance, the South African Environmental Justice Networking Forum asserted (1997, quoted in McDonald 2002) “Environmental justice is about social transformation directed towards meeting basic human needs and enhancing our quality of life—economic quality, health care, housing, human rights, environmental protection, and democracy. In linking environmental and social justice issues, the environmental justice approach seeks to challenge the abuse of power which results in poor people having to suffer the effects of environmental damage caused by the greed of others”.

How can such environmental justice movements achieve such claimed transitions towards more sustainable futures? Several strands can be distinguished that are useful for delineating their potential roles for sustainability transitions. The distinction posed by Gadgil and Guha (1993) between *intramodal* and *intermodal* ecological conflicts is helpful in this regard. Intramodal conflicts emerge over the distribution of environmental benefits and burdens *within an established pattern of resource use between and amongst different social groups*, sometimes along class, gender or ethnic lines. For example, this entails conflicts between farmers over the distribution of irrigation water; access to common land; or exploitation quotas (González de Molina et al. 2009). It also covers conflicts over equitable distribution of other environmental benefits and burdens (water, energy, parks and green spaces, land, etc.) across the same user group. Related movements may be arguing for a reduction of environmental hazards through improved governance or technology, together with a more equitable distribution of environmental goods and bads (ibid). Some of them might also take the form of NIMBY conflicts, concerned mainly of not having hazardous project in their own backyard, but without fundamentally questioning the underlying systems and their potential (un)sustainability. This type of ecological distribution conflict is unlikely to contribute directly to radical transformations in socio-metabolic configurations, as they often focus only on specific places and do not question the mode of production itself. However, if redistributive claims are accomplished and environmental cost-shifting is diminished as a result, this could lead to improved management within a given socio-metabolic configuration.

On the contrary, intermodal conflicts are those which *defend a particular mode of resource use against industrial society’s attempts to transform it*. González de Molina et al. (2009) give as a historical example, the case of Galician farmers (Spain) who fought to preserve communal land from attempts of industrialization. In doing so, they played a key role for maintaining an agricultural model largely independent from fossil energy. A current example is the Prey Lang Community Network in Cambodia, a forest movement that originated to protect one of the biggest primary forests in Southeast Asia. For decades, Prey Lang forest has been under threat of logging and contamination due to illegal timber trade, agro-industries and mining concessions. After years of cooperation between forest-depended communities to halt forest destruction, the network was established in 2007 by local activists of Khmer and Kuy indigenous identity. The decentralized movement, spanning several provinces, established regular community forest patrols to stop illegal loggers, burned illicit timber piles, confiscated chain saws, lobbied authorities and launched several campaigns that draw wide attention to their cause. In 2012, following increasing awareness and pressure before general elections, the government cancelled several extractive projects jeopardizing the forest. Some described this as a ‘rare victory’ (EJAtlas 2015b). In 2015, the movement was awarded the UNDP Equator Prize that recognizes *“*outstanding local achievement in advancing sustainable development”^6^.

A powerful global example of how grassroots movements can shape sustainability processes is also given by transnational agrarian movements, such as La Via Campesina (LVC), or the International Planning Committee for Food Sovereignty (IPC). In their defense of peasant agriculture and against large-scale capitalist industrial agriculture, both LVC and the IPC have fundamentally contributed to promoting agroecology as a sustainable agriculture model across the globe. Also, their efforts in making education accessible to poor groups, thanks to popular peasant universities, represent an important contribution to sustainability efforts (Edelman and Borras 2016).

Movements arising out of intermodal conflicts may take the form of groups confronting specific forms of damaging industrial activities as well as those claiming against unknown risks (Beck 1992). Yet their scope of action goes often well beyond specific places and feeds into alliances and solidarity with other movements across regions and the globe (see Tramel 2016). It is a type of environmentalism that is different from conservationism focusing on wildlife and also from ecological modernization focusing on technological change and on the internalization of externalities in the price system. As capitalism is a major force behind the expansion of extractivist, industrial projects that transform former socio-metabolic configurations across the globe, intermodal movements, either implicitly or explicitly, tend to take anti-capitalist stances^7^. Such movements often question the dominant form of valuation of resource uses (i.e. monetary values and cost-benefit analyses) and renegotiate the values deemed relevant for sustainability (Martinez-Alier 2002). Sometimes, particularly when the resistance weakens, demands for monetary compensation are made (in a framework of ‘weak sustainability’; Martinez-Alier et al. 1998). The same groups, at other times or when feeling stronger, might argue in terms of values which are not commensurate with money, such as indigenous territorial rights, irreversible ecological values, human right to health or the sacredness of Mother Earth, implicitly defending a conception of ‘strong sustainability’. In contesting and redefining the very economic, ecological and social principles behind particular uses of the environment, such intermodal conflicts are those that are most clearly forces towards broader sustainability transitions.

Whether ‘just sustainabilities’ (Agyeman and Evans 2004) are really easy to achieve has been forcefully questioned by Andrew Dobson (1998), who pointed to the conflicts and tensions between environmental sustainability and distributive justice, both widely regarded desirable social objectives. Let us consider ‘climate justice’. Removing world’s energy poverty by providing every citizen with a right to burn fossil fuels to the tune of emitting 5 tons of CO_2_/year could be seen as a modest and equitable outcome in distributive terms—but it would not be conducive to sustainability. The sustainability condition would argue that the European average of 10 tons of CO_2_/person/year is far too high and should be reduced quickly by 70 or 80%. Removing energy poverty is desirable but cannot entail raising the world average to 5 tons/person/year. Other means must be sought, such as alternative sources of energy perhaps financed by the ‘ecological/carbon debt’ owed historically by the rich (Warlenius et al. 2015). Acknowledgement of liability for climate change (brutally excluded in the Paris COP agreement of 2015) would mean a redistribution of wealth among and within nations. However, Dobson’s point remains that distributive ‘climate justice’ in itself does not ensure sustainability, or rather ‘climate justice’ implies two separate objectives, one regarding equity and another one regarding climate stability.

In practice, by looking at the outcomes of different ecological distribution conflicts collected in the EJAtlas, we could give many examples in which both objectives are served; hence, in which the success in environmental justice does not undermine the objective of sustainability, rather on the contrary. For instance, the proposed Fuleni coal mine in Kwa Zulu Natal stands very near the border of the very valuable Hluhluwe-Mfolozi Wilderness area. There is confluence of protests from conservationists and the local people (in MCEJO - Mfolozi Community Environmental Justice Organisation) opposing mining. Although their main motivations are local, both conservationists and local people have learnt to praise the policy of ‘leaving coal in the hole’ against climate change (EJAtlas 2016b). In Sompeta in Andhra Pradesh, the government had allotted 972 acres of land including wetlands to Nagarjuna Construction Company to build a coal-based thermal power plant. Community members were extremely opposed to the construction since it would destroy their entire livelihoods, which is based on this land to sustain their fisheries and farmlands. They allied with environmentalists and after 8 years of strong resistance, they were successful in 2015 in stopping the project. Now, there is some local implementation of alternative energy systems (EJAtlas 2015c).

Many similar stories can be found in the EJAtlas^8^. They illustrate indeed our hypothetical rule: more success for environmental justice, more environmental sustainability.

### Sustainability transitions reshape socio-metabolic configurations

All visions of sustainability transitions entail concomitant transformations in socio-metabolic relations. Nowadays, the primary emphasis in socio-metabolic terms is the transition to a low-carbon and resource-efficient economy. This calls for major changes in energy, transport, and agri-food systems (Geels 2012), a fundamental transformation towards more sustainable modes of production and consumption (Markard et al. 2012) and re-localization of production and consumption to shorten resource flow and supply chains (Asara et al. 2015).

Yet, a narrow focus on increased efficiency, or *relative* dematerialization and decarbonization, is insufficient, not least because it might lead to Jevons’ effects (i.e. increase in efficiency might lead to greater, rather than lesser, total consumption), and many argue for a more radical transformation of the socio-metabolic regime (Polimeni et al. 2008). Attention to the many social, ecological and economic issues of sustainability is required. Furthermore, if we conceptualize a major sustainability transformation as a shift into a completely new socio-metabolic regime, it becomes clear that this time the transition must entail a substantial reduction in energy and material flows per capita (Fischer-Kowalski and Rotmans 2009). This is in sharp contrast to past transitions which were associated with a substantial increase in metabolic rates. This thermo-dynamic reality is what leads Degrowth, *Décroissance* or *Post-Wachstum* proponents to mobilize for social transformation towards absolute reductions of energy and material throughput; as well as more equitable distribution of resources, as a means to combine social justice and environmental concerns (Demaria et al. 2013).

This is uncharted territory, calling for a shift to a yet unknown type of social organization. Such a transition can be well informed by combining socio-metabolic assessments with a political economy/ecology analysis of how particular forms of technology and resource use regimes are constructed and employed, who owns the resources and how benefits are distributed; and how movements of opposition contest and aim to reshape resource governance. Take for example the transition from fossil to renewable energy sources. Biofuels can be produced at the local level in a decentralized and democratic manner with waste materials. They can also be produced on a large-scale based on environmentally destructive monocultures that are far from resolving the problem of energy supply (Giampietro and Mayumi 2009), but rather dispossess local farmers through associated land-grabbing (Borras et al. 2010; Scheidel and Sorman 2012). In the case of the latter, such mistakenly called ‘sustainability transitions’ would just produce new socio-metabolic configurations that are as conflictive and unsustainable as the previous, restarting the circle outlined in Fig. 1.

But there are also historic cases in which sustainability transitions pushed new socio-metabolic configurations that did not (immediately) provoke a new set of unsustainabilities, conflicts and mobilizations. Bond and Dorsey (2010) put forward as an example the 1996 Montreal Protocol on chlorofluorocarbons (CFCs) which succeeded in banning emissions outright to prevent growth of the hole in the ozone layer, as perhaps the last example of effective globally coordinated top-down environmental action. In the EJAtlas, we also find numerous cases of effective activism from below leading to reduced extractive activities or moratoria at the project, local, sub-national and national scale. The decline of the shale gas boom in Europe is one notable example, with countries such as France, Bulgaria and the Netherlands, among others, declaring a ban on the exploitation of new natural gas deposits (EJAtlas 2015d). It should be noted, however, that while extraction is not proceeding in these countries, pipeline connectivity to import fracked gas from North Africa and other locations is expanding, potentially shifting conflicts elsewhere. But opposition also appears there (EJAtlas 2015e).

We may also note that the way sustainability transitions reshape socio-metabolic configurations depends on the materiality of resources themselves and how these contribute to shaping power relations and social systems. For example, oil as a resource requires large-scale capital investment and centralized control and distribution. In contrast, many renewable energies such as wind and solar could be harnessed at small-scales with lower capital investment, meaning they could be controlled at the community scale with important implications for decentralized and democratic governance (Lawhon and Murphy 2012). But also here, wind-energy is often produced at large scale and can lead to local conflicts on land use or biodiversity conservation (Avila, this feature). This points to how within low-carbon metabolic configurations, environmental justice activists aim to bring attention to issues of scale, control, sovereignty and democracy, arguing that the sustainability transformation must be defined not only by changes in resource use, i.e. a shift from fossil to renewables, but also in how they are governed. For instance, the Lubicon Cree Community of Little Buffalo, Alberta, who have suffered from massive oil spills and contamination related to tar sands exploitation on their territory have recently launched the Piitapan Solar Project that powers the health center as a means of resistance to tar sands expansion through showing the world that the shift to renewables is possible (EJAtlas 2014c). This highlights that energy transitions and environmentally just socio-metabolic configurations are not only about the form of energy, but about energy for what and for whom and under what social relations.

## Breaking the vicious cycle of unsustainabilities and ecological distribution conflicts

So far we have addressed some key linkages between socio-metabolic configurations, ecological distribution conflicts, environmental justice movements and sustainability transitions. As seen in the previous section, some transitions bring an end to some ecological distribution conflicts, but they also can produce a whole set of new ones.

For instance, Špirić, this feature, and Pérez-Rincón et al. this feature offer a historic account on how ecological distribution conflicts change across different political and economic regime transitions. Above, we have mentioned biofuels and land-grabbing conflicts as an example of how sustainability transitions can ironically trigger a whole new set of unsustainabilities and conflicts. The EJAtlas reports numerous of such cases, but also here, local movements have managed to stop many unsustainable agro-fuel projects (Temper 2018). Many other examples exist in which renewable energy systems have caused new conflicts and mobilizations (for hydroelectric dams see Del Bene, et al., this feature; for windfarms, Avila, this feature; for waste to energy see Herrero and Vilella, this feature, as well as Johnson et al., this feature). Further examples also include the recent emergence of ‘green grabs’ (Fairhead et al. 2012), in which resources are grabbed from local users for environmental ends such as for CO_2_ sequestration through large-scale forestry projects (Lyons and Westoby 2014).

Hence, as sustainability transitions move on to resolve old issues, they create new problems along the way by altering socio-metabolic configurations that—again—re-distribute environmental benefits and burdens. Sieferle and Müller-Herold (1996) argued that a ‘risk spiral’ exists in sustainability, in which the reduction of one risk usually requires innovations that produce new uncertainties and future sustainability problems. In our analysis, we see this unfolding as a ‘conflict spiral’ in which the solution of former sustainability issues creates new environmental conflicts through a redistribution of environmental benefits and burdens. Is there a way to escape this conflict spiral?

Progress, at least, requires reducing rather than expanding the circles of this conflict spiral across resource use regimes and to avoid that new pressures are not shifted to marginalized groups, such as indigenous. Sustainability politics are needed that consider impacts beyond narrow fixes to single problems but rather across different resource use regimes, by anticipating the social and ecological implications of proposed socio-metabolic configurations across different social groups. In line with our hypothesis, we are convinced that this calls for a reduction of social metabolism in absolute terms, particularly of those material and energy flows that are most damaging and conflictive. The Degrowth movement, composed not only of academics but also activists, has collected many ideas of how this may be envisioned and achieved (D’Alisa et al. 2015). With no doubt, it would require a fundamental restructuring of the way modern societies operate.

To achieve such restructuring, co-production of knowledge and exploration of alternatives is strongly needed and environmental justice movements, in alliance with other movements, have much to contribute here (Martinez-Alier 2012; Conde 2014; Kothari et al. 2015; Temper and Del Bene 2016). Beyond this, environmental justice movements are also crucial in monitoring impacts of new socio-metabolic configurations provoked by emerging alternatives. Karl Polanyi (1944) argued that a double movement exists, meaning a dialectical process of marketisation and push for social protection against that marketisation. Here, we see that a double movement exists where environmental justice movements react to socio-metabolic configurations that are unsustainable in either their biophysical characteristics or governance. In defense of their means of existence and subsistence, but also for the general interest of protecting the public good, environmental justice movements are crucial in politicizing and sometimes also transforming such unsustainabilities. They continually contribute to reframing and questioning what sustainability means, which vision of sustainability is operationalized, and what socio-metabolic configuration is most compatible with social justice and ecological health. Environmental justice movements are, therefore, essential ‘safeguards of society’ that address adverse impacts of not only unsustainable policies, but also the impacts of sustainability policies themselves. Therefore, they might be among the most promising social forces to promote sustainability. There it is where sustainability science should be looking for alliances to achieve change.

## Conclusions

This paper has aimed to address a fundamental paradox of sustainability. On one hand, science has been consolidating the arguments to prove that humanity is facing a sustainability crisis, yet on the other, calls for action seem to have been futile. Scientists might get the feeling that their voices have not been heard, but instead here we argue that it might be them who failed to hear the voices of those who struggle everyday for sustainability, even at the expense of their own lives.

With the conceptual framework laid out in this paper, we have aimed to give a systematic overview and clarify how struggles over environmental conflicts can contribute to processes towards sustainability. Driven by patterns of unsustainable social metabolism, ecological distribution conflicts often provoke the emergence of environmental justice movements. Their collective actions to shed light on—and to transform—these resources uses damaging humans and the environment can contribute to transitions towards more sustainable futures in various ways that we discussed in this paper. From this perspective, conflicts bear a tremendous power of mobilizing social forces for change.

The Environmental Justice Atlas and other inventories, such as those of OCMAL (*Observatorio de Conflictos Mineros de América Latina*) or GAIA (Global Alliance for Incineration Alternatives) show that there are thousands of local environmental conflicts where millions of people struggle to defend their health and livelihood. While not only contributing to the sustainability of the economy by transforming environmental injustices caused by unsustainabilities, such environmental justice movements are at the forefront in repoliticizing and reimagining sustainability transitions. This is urgently needed to confront the profound sustainability crises of today.

Contributions of environmental frontline defenders are slowly reaching more global visibility, such as through the Goldman environmental prize, also known as ‘green Nobel’, or the UNDP Equator prize awarding community-based initiatives for sustainability. Nevertheless, environmental activists are coming under increasing threat and repression. Violence against them has become systematic. Alliances for sustainability must, therefore, not only integrate in a fruitful way the work of academics and activists—for example through co-produced knowledge—but also seek growing institutional support for threatened grassroots activists. How such mechanisms of support and protection may look like in practice, remains to be explored. Relevance of developing such effective support is high as currently many of them are not only essential but also endangered actors for sustainability.

## Footnotes

1 For an overview of the intellectual history of the concept and its relation to the social sciences see Fischer-Kowalski (1997, 1998), Fischer-Kowalski and Hüttler (1999).

2
http://www.ejnet.org/ej/principles.html.

3 There are for instance activist groups with names including the words ‘environmental justice’ in Sri Lanka (Camisani, this feature), Mozambique, or Brazil (da Rocha et al., this feature). Similar groups use other names elsewhere, such as Acción Ecológica in Ecuador, CENSAT in Colombia, and so many others, some of them grouped in Friends of the Earth International.

4 For a discussion on what ‘environmental justice success’ can mean, see Özkaynak et al. (2015).

5 Based on EJAtlas data, July 2017. The remaining 4% are comprised of cases with not visible mobilizations, or unknown start of mobilization.

6
http://www.equatorinitiative.org/.

7 In this way, we can argue that *intermodal* movements go beyond simply tweaking capitalism to be greener (which may apply to some *intramodal* movements). By contesting the very socio-metabolic basis of the industrial capitalist growth economy, they may therefore also go beyond mitigating the ‘second contradiction of capitalism’ provoked by environmental destruction (O’Connor 1988). We may argue that such uncompromising, intermodal resistance serve to threaten the very basis of the capitalist economy itself. They may thus represent what O’Connor (1988***, 28) described as the “powerful social movements demanding an end to ecological exploitation”.

8 For instance in Phulbari, Bangladesh, there was very violent repression with several victims leading to a ban on open-pit coal mining in the area and withdrawal of international funding (https://EJAtlas.org/conflict/protest-against-open-pit-coal-mine-project-in-phulbari-region). Also the Ende Gelände movement in Germany, motivated not only by climate change, became an important force in reducing or stopping lignite mining in the country (https://EJAtlas.org/conflict/linginte-mining-and-the-ende-gelande-movement).
